# Supplementary material for: Empirical methods for controlling false positives and estimating confidence in ChIP-Seq peaks
Source: BMC Bioinformatics. 2008 Dec 5;9:523. doi: 10.1186/1471-2105-9-523 (PMC2628906; doi:10.1186/1471-2105-9-523)
Supplement: Additional File 1 — A variety of html documents from the USeq web site detailing the available applications, their best usage, output file type descriptions, command line menus, etc. [file 1471-2105-9-523-S1.zip › USeqUserGuides/JavaDoc.zip!/JavaDoc/gata/geneGlyphs/GeneRepGlyph.html]

GeneRepGlyph


|  |  |  |
| --- | --- | --- |
| **Overview** \| **Package** \| **Class** \| **Use** \| **Tree** \| **Deprecated** \| **Index** \| **Help** | |  |
| **PREV CLASS**   **NEXT CLASS** | **FRAMES**    **NO FRAMES**     **All Classes** |  |
| SUMMARY: NESTED \| FIELD \| CONSTR \| METHOD | DETAIL: FIELD \| CONSTR \| METHOD |  |


---


## gata.geneGlyphs Class GeneRepGlyph

```
java.lang.Object
  gata.geneGlyphs.GeneRepGlyph
```

---

``` public class GeneRepGlyph extends java.lang.Object ```

**Author:**
:   Nix
    Shape representation for a GeneRep- the DNA component of genomic annotation.

---

**Constructor Summary**

`GeneRepGlyph(GeneRep geneRep,
GATAParams gataParams,
AnnoSpecParams annoParams)`


| **Method Summary** | |
| --- | --- |
| `void` | `draw(double yCoordinate)` |
| `void` | `drawTransGrpGlyphs()` |
| `java.awt.geom.Rectangle2D.Double` | `getBoundingBox()` |
| `int` | `getEnd()` |
| `double` | `getNonOverlapYCoor()` |
| `int` | `getStart()` |
| `void` | `setTransGrpGlyphs(java.util.ArrayList transGrpGlyphs)` |
| `java.lang.String` | `toString()` |

**Methods inherited from class java.lang.Object**

`equals, getClass, hashCode, notify, notifyAll, wait, wait, wait`

**Constructor Detail**

### GeneRepGlyph

```
public GeneRepGlyph(GeneRep geneRep,
                    GATAParams gataParams,
                    AnnoSpecParams annoParams)
```


**Method Detail**

### toString

```
public java.lang.String toString()
```

:   **Overrides:**: `toString` in class `java.lang.Object`

---


### draw

```
public void draw(double yCoordinate)
```

---


### drawTransGrpGlyphs

```
public void drawTransGrpGlyphs()
```

---


### setTransGrpGlyphs

```
public void setTransGrpGlyphs(java.util.ArrayList transGrpGlyphs)
```

---


### getNonOverlapYCoor

```
public double getNonOverlapYCoor()
```

---


### getBoundingBox

```
public java.awt.geom.Rectangle2D.Double getBoundingBox()
```

---


### getEnd

```
public int getEnd()
```

---


### getStart

```
public int getStart()
```


---


|  |  |  |
| --- | --- | --- |
| **Overview** \| **Package** \| **Class** \| **Use** \| **Tree** \| **Deprecated** \| **Index** \| **Help** | |  |
| **PREV CLASS**   **NEXT CLASS** | **FRAMES**    **NO FRAMES**     **All Classes** |  |
| SUMMARY: NESTED \| FIELD \| CONSTR \| METHOD | DETAIL: FIELD \| CONSTR \| METHOD |  |


---
